# Oral Management Improves Patient Outcomes in Hematopoietic Stem Cell Transplantation

**DOI:** 10.1016/j.identj.2025.04.003

**Published:** 2025-05-09

**Authors:** Mutsuko Moriwaki, Mikayo Toba, Makiko Takizawa, Hiroaki Shimizu, Haruna Tanaka, Chihiro Takahashi, Shinobu Imai, Masayuki Kakehashi, Kiyohide Fushimi

**Affiliations:** a Quality Management Center, Institute of Science Tokyo, Bunkyo-ku, Tokyo, Japan; b Saitama Medical University General Medical Center, Kawagoe, Saitama, Japan; c Tokyo Metropolitan Cancer and Infectious Diseases Center Komagome Hospital, Bunkyo-ku, Tokyo, Japan; d Department of Nursing, Institute of Science Tokyo Hospital, Bunkyo-ku, Tokyo, Japan; e Department of Pharmacoepidemiology, Showa University Graduate School of Pharmacy, Shinagawa, Tokyo, Japan; f Graduate School of Biomedical and Health Sciences, Hiroshima University, Hiroshima City, Hiroshima, Japan; g Graduate School of Medical and Dental Sciences, Institute of Science Tokyo, Bunkyo-ku, Tokyo, Japan

**Keywords:** Hematopoietic stem cell transplantation, Oral management, Antibiotic, Narcotic, In-hospital mortality, Dental hygiene

## Abstract

**Introduction and aims:**

Oral bacteria influence bloodstream infections in hematopoietic stem cell transplantation (HSCT). We investigated the effects of oral health management and its relationship with medical care delivery systems.

**Methods:**

Patients aged >16 years who underwent HSCT, discharged from Japanese acute care hospitals between April 2018 and March 2022, were categorized into autologous and allogeneic HSCT groups. Multivariable analysis assessed the impact of peri-HSCT oral management on antibiotic use, narcotic injections, and mortality rates.

**Results:**

We included 12,248 patients, 5936 autologous and 6312 allogeneic HSCT patients, across 298 hospitals. The defined daily dose (DDD) of antibiotic use within 14 days post-transplantation in the oral and nonoral management groups for allogeneic HSCT patients was 34.10 (standard deviation [SD] 20.35) vs 36.37 (SD 21.33); broad-spectrum antibiotics use was 23.87 (SD 15.82) vs 24.45 (SD 15.76). Within 30 days post-transplantation, the DDD of antibiotic use was 69.13 (SD 40.18) vs 75.16 (SD 43.47) was 45.70 (SD 29.63) vs 47.95 (SD 30.48), respectively. In allogeneic HSCT patients, oral management resulted in lower DDD of antibiotic use by 2.66 within 14 days and 6.74 within 30 days post-transplantation, after adjustment for relevant factors. Broad-spectrum antibiotic use within 30 days post-transplantation showed a lower DDD by 2.79 (*P* < .01). Narcotic use led to a 0.34 lower DDD (*P* < .01) within 14 days and 0.70 lower DDD (*P* < .01) within 30 days. In autologous HSCT patients, oral management did not affect the outcomes. The certification standard for unrelated HSCT, categorized into four classes (no certification and certification levels 1-3), was associated with an 8.41 point increase in hospital oral management implementation per class.

**Conclusion:**

Ensuring an appropriate oral environment for allogeneic HSCT patients helps preventing infection, extending life expectancy, and alleviating pain.

**Clinical relevance:**

Coordinated care between dental and medical teams is essential to deliver safe, personalized, and high-quality patient outcomes during HSCT.

## Introduction

Hematopoietic stem cell transplantation (HSCT) is a highly efficacious treatment modality for various hematologic malignancies, including leukaemia, lymphoma, and multiple myeloma. Data compiled by the Japanese Data Center for Hematopoietic Cell Transplantation revealed an increase in transplant procedures, from 2662 cases in 1991 to 5853 in 2021, marking a more than two-fold increase over three decades.^1^ In 2021, the 1-year transplant survival rate surpassed 90% and 80% for autologous and allogeneic HSCTs, respectively, demonstrating a continuous upward trend. HSCT plays a pivotal role in prolonging patient survival by building on the positive outcomes achieved through previous treatment approaches.^2^

HSCT complications include issues arising from conditioning regimens, immune-related challenges, and late post-transplantation effects. The toxicity associated with the conditioning regimen includes myelosuppression and stomatitis and leads to susceptibility to infections and symptoms such as nausea, vomiting, and diarrhoea. Infections during the myelosuppressive phase may escalate to sepsis, whereas bloodstream infections (BSI) subsequent to HSCT are correlated with elevated mortality rates.^3^ The incidence of BSI in the weeks following HSCT ranges from 12% to 75%.^4^ There are also reports that the distribution of microorganisms obtained from the blood of patients with BSI during allogeneic HSCT resembles the distribution of microorganisms from oral cultures.^5^ Cappellano et al^6^ reported that approximately 21% of patients who underwent HSCT experienced BSI within 30 days of the procedure, with a mortality rate of 28% by day 180 post-transplantation. The cumulative mortality risk for patients with BSI was significantly higher than that for other patients. Acute graft-versus-host disease occurs early after allogeneic HSCT.^7^ This disease is characterized by symptoms encompassing skin manifestations, gastrointestinal issues, and liver dysfunction. Anticancer medications such as methotrexate are employed as preventive measures against graft-versus-host disease, although they exacerbate the onset of oral mucositis (OM).^7^

OM commonly arises in numerous patients because of the adverse effects of conditioning,^8^, ^9^, ^10^, ^11^, ^12^ with reports indicating that OM develops in up to 80% of patients.^13^ OM can also lead to infections that result in death in certain instances.^14^ It is also often associated with severe pain and extensive ulceration, occasionally necessitating opioid analgesics, thereby affecting eating habits and purportedly diminishing the patient’s quality of life.^8^^,^^10^

Ensuring a healthy oral cavity in patients undergoing HSCT significantly influences their prognosis by aiding infection prevention and enhancing their quality of life, particularly in pain management and nutrition. Professional oral management, primarily led by dentists, is increasingly being recognized as vital. As part of fundamental oral care, a comprehensive oral assessment was conducted 2 weeks before transplantation. Interventions such as tooth extraction, periodontal therapy, and tartar removal are recommended based on the oral condition.

In Japan, a perioperative oral management fee was introduced in 2016 for a comprehensive series of pre- and post-treatment oral management procedures in collaboration with dentists and cancer-treatment doctors. The objective was to mitigate complications, such as aspiration pneumonia, among patients undergoing cancer treatments, such as radiotherapy or chemotherapy, as well as postoperatively. Consequently, patients who have undergone oral management are documented in the clinical performance data, facilitating the evaluation of patient outcomes through large-scale database analysis.

Previous studies have elucidated the incidence of OM resulting from oral management in patients undergoing HSCT; however, the sample sizes of these studies were relatively limited.^15^, ^16^, ^17^ Although oral management before and after transplantation in HSCT is theoretically efficacious, randomized controlled trials demonstrating its effectiveness are scarce, with outcomes often deviating from anticipated results.^11^^,^^18^ The Japanese Society for Transplantation and Cellular Therapy points out that the importance of oral management in HSCT is so fundamental and widely recognized that it is ethically impossible to conduct clinical studies with negative control. As a result, there is a lack of high-level evidence to support the guidelines. Therefore, further research with stronger evidence is required.^19^

Therefore, to investigate the effects of pretransplantation oral management on infection and OM in patients undergoing HSCT, we used multicentre data to clarify the short-term effects and types of medical care delivery systems that influence the implementation of oral management.

## Methods

### Aim, design, and setting

We conducted a retrospective study using data from acute care hospitals. We aimed to elucidate the short-term effects and assess the influence of a medical care delivery system on oral management.

In this study, we utilized the Diagnosis Procedure Combination (DPC) database. The DPC is a patient classification system for acute inpatients that originated in Japan and is designed to enhance transparency and visibility in acute-stage medical care. These systems were established in 2003 by the Ministry of Health, Labour, and Welfare as a comprehensive payment mechanism and were employed for acute hospital care and medical resource allocation. Acute care hospitals in Japan participate in this system and report information regarding medical procedures to the government.^20^ As of 2020, the DPC database covers 1757 facilities and 483,180 beds, accounting for 24.5% of general hospitals and 54.4% of the number of beds in Japan. The DPC database collected the following information: disease name information linked to the International Classification of Diseases 10 codes, such as patient age and sex, main diagnoses, pre-existing comorbidities, postadmission complications, dates of admission, and discharge, route of admission, discharge destination, outcome at discharge, and surgical procedures. Several studies on data reliability and clinical epidemiology have been conducted using this database.^21^, ^22^, ^23^

### Study population

The study cohort comprised patients aged 16 years and older who were admitted to hospitals and underwent HSCT between 1 April 2018, and 31 March 2022. Exclusion criteria included patients who underwent any procedure (including transplantation) other than HSCT during hospitalization, those who underwent allogeneic and autologous HSCT within a single hospitalization episode, and those with outlier antibiotic and narcotic usage amounts. In Japan, if oral management is performed by a dentist up to 1 month before HSCT, medical fees can be claimed. In this study, patients for whom this claim was made were categorized as the ‘oral management group’. Pretransplantation oral management may include oral cleaning, tartar removal, periodontal treatment, tooth extraction, and denture adjustment, depending on the patient’s oral condition. Since the DPC data are retrieved from medical treatment records, the specific details of the treatment are not included in this medical fee claim information. However, oral management is conducted based on a physician’s request to a dentist for oral management in preparation for HSCT, and it is assumed that treatment is tailored to each patient’s condition. This variable was established to distinguish between patients who received an oral health examination by a dentist and those who did not before undergoing HSCT.

### Outcome

In this study, the outcomes evaluated were the use of antibiotics (administered via injection) and narcotics (administered via injection) at 14 and 30 days after HSCT and in-hospital mortality rates. Antibiotics were categorized according to the Anatomical Therapeutic Chemical Classification System established by the World Health Organization Collaborating Center for Drug Statistics Methodology. Antibiotics included all antibiotic agents and broad-spectrum antibiotics, including tazobactam/piperacillin, fourth-generation cephalosporins, carbapenems, and injectable fluoroquinolones (Table S1). Drug doses were standardized according to the defined daily dose (DDD).^24^

### Variables

The following variables were used in this study: patient factors, including age, sex, length of hospital stay, Charlson comorbidity index (CCI),^25^^,^^26^ body mass index (BMI), transfer admission, and death. Various hospital factors were considered variables, including academic hospital status, attached dental clinic, designation as a cancer centre hospital, availability of nursing staff, case volume of HSCTs at each hospital, and allocation of staff dedicated to HSCTs (doctors, nurses, and hematopoietic cell transplant coordinators [HCTC]). The classification system for certification standards for performing HSCTs between unrelated individuals, as defined by the Japanese Society for Transplantation and Cellular Therapy, was employed to assess the allocation of specialized staff for HSCTs. This certification standard was stratified into three classes (Classes 1, 2, and 3). The Transplant Facility Accreditation Committee of the Japanese Society for Transplantation and Cellular Therapy accredited transplant facilities. The criteria for certification were developed in collaboration with the Japan Marrow Donor Program, Japanese Data Center for Hematopoietic Cell Transplantation, and Japanese Red Cross Society (an organization supporting hematopoietic stem cell donation). These established requirements indicate that facilities performing transplantation using hematopoietic stem cells from unrelated individuals should meet the transplant facilities, composition, and staffing of the transplant team, transplant outcomes, and other relevant aspects.

Class 1 refers to medical institutions (medical departments) that fulfil all certification standards. Class 2 designates medical institutions (medical departments) that meet all certification criteria, with the exception of transplant certification, nursing, and HCTC. Class 3 signifies low-volume centres that satisfy all certification standards except for transplant certification, nursing, and HCTC.

### Statistical analyses

Patients were stratified into groups for each variable based on the presence or absence of oral management before and after autologous and allogeneic HSCT. Comparative analyses between these groups were performed using appropriate statistical tests, such as the Mann–Whitney *U* and Chi-square tests. Subsequently, multiple regression analyses were performed with antibiotic usage status, narcotic usage status, and in-hospital death as dependent variables. Moreover, a generalized estimating equation model was employed to accommodate the hierarchical structure of the data nested within the hospitals.

Next, we aggregated data on patients undergoing autologous and allogeneic HSCT at the hospitals and compared the oral care implementation rates of hospitals by medical care delivery system (academic hospital, attached dental clinic, cancer centre hospital, nursing staffing, bed size, case size of HSCTs at each hospital, and placement of specialized staff related to HSCTs) (Mann–Whitney *U* and Kruskal–Wallis tests). Next, we conducted a multiple regression analysis of the medical care delivery system that influenced oral management implementation rates in hospitals.

IBM SPSS Statistics for Windows, version 29 (IBM Corp.), was used in all analyses, and the significance level was set at 5%.

### Ethical considerations

This study was approved by the Medical Research Ethics Committee of Institute of Science Tokyo (approval number: M2000-788). The requirement for informed consent was waived by the Ethical Review Board as all personal information was excluded, and the deidentified data was sent to the researchers only for secondary use.

## Results

The study analysed 12,248 patients, including 5936 who underwent autologous HSCT and 6312 who underwent allogeneic HSCT, across 298 hospitals (Figure). Among the patients undergoing autologous HSCT, 111 (1.9%) had acute leukaemia, 2628 (44.3%) had non-Hodgkin’s disease, 206 (3.5%) had Hodgkin’s disease, and 2989 (50.4%) had multiple myeloma/immune system malignant neoplasms. In total, 1317 individuals (22.19%) were in the oral care group. The comparison of background factors between the two groups, with and without oral management, revealed similar proportions of men (57.5% vs 57.8%, *P* = .83), mean ages (57.6 years [standard deviation (SD) 10.3] vs 57.7 years [SD 10.1], *P* = .88), and lengths of hospital stay (35.5 days [SD 16.1] vs 36.4 days [SD 18.2], *P* = .69).

**Fig:**
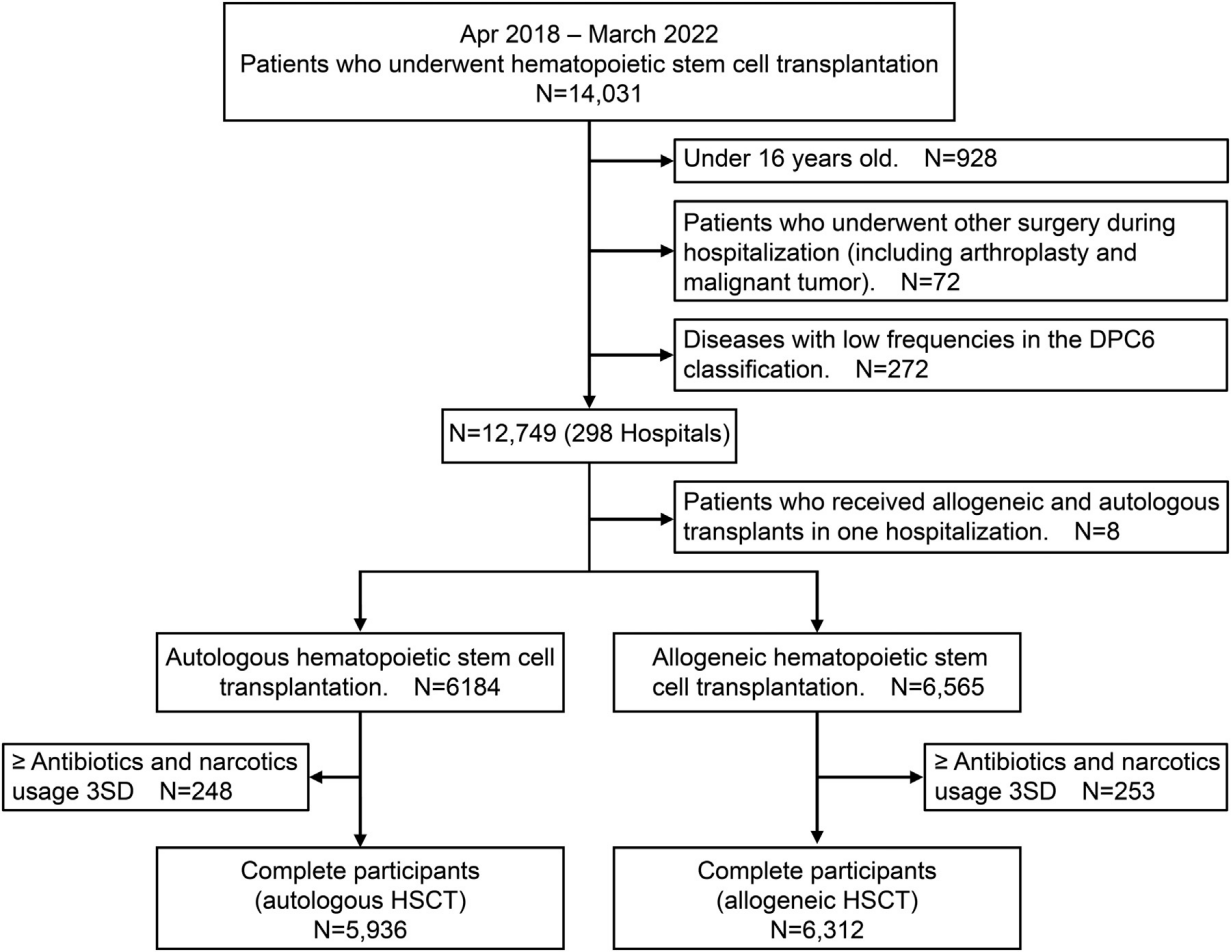
The participant flow for the study.

Among the patients undergoing allogeneic HSCT, 3659 (58.0%) had acute leukaemia, 1035 (16.4%) had non-Hodgkin’s disease, 1036 (16.4%) had myelodysplastic syndromes, 311 (4.93%) had myeloproliferative neoplasms, and 133 (2.11%) had aplastic anaemia. Additionally, 2865 individuals (45.49%) were in the oral management group among allogeneic group patients. Comparing patient background factors between the two groups, with and without oral management, revealed similar proportions of men (58.2% vs 59.0%, *P* = .49), mean ages (50.6 years [SD 14.1] vs 49.8 years [SD 14.1], *P* = .02), and lengths of hospital stay (86.4 days [SD 38.0] vs 91.6 days [SD 42.3], *P* < .01) (Table 1).

**Table 1:** Characteristics of the participants.

	Autologous hematopoietic stem cell transplantation							Allogeneic hematopoietic stem cell transplantation						
	Total *N* = 5936		Oral management *N* = 1317		Nonoral management *N* = 4619		P*	Total *N* = 6312		Oral management *N* = 2865		Nonoral management *N* = 3447		P*
《Patient factor》														
Men, *n*, %	3427.00	57.73	757.00	57.48	2670.00	57.80	.83	3700.00	58.62	1666.00	58.15	2034.00	59.01	.49
Age, mean, SD	57.65	10.18	57.58	10.31	57.67	10.14	.88	50.18	14.10	50.60	14.10	49.83	14.10	.02
Transfer (admission date)							.81							.64
Fully assisted, *n*, %	11.00	0.19	3.00	0.23	8.00	0.17		22.00	0.35	8.00	0.28	14.00	0.41	
Partly assisted, *n*, %	47.00	0.79	9.00	0.68	38.00	0.82		39.00	0.62	19.00	0.66	20.00	0.58	
Without assistance, *n*, %	5878.00	99.02	1305.00	99.09	4573.00	99.00		6251.00	99.03	2838.00	99.06	3413.00	99.01	
BMI	22.94	3.70	23.00	3.69	22.92	3.71	.51	21.90	4.19	21.97	4.71	21.85	3.71	.11
CCI score, mean, SD†	2.20	1.02	2.20	1.03	2.19	0.05	.63	1.01	1.30	1.00	1.27	1.02	1.33	.80
Total body irradiation	3.00	0.05	3.00	0.23	3.00	0.06	.10	3768.00	59.70	1637.00	57.14	2131.00	61.82	<.01
Most resource-consuming diagnosis							.81							.06
Acute leukaemia, *n*, %	111.00	1.87	27.00	2.05	84.00	1.82		3659.00	57.97	1615.00	56.37	2044.00	59.30	
Non-Hodgkin’s lymphoma, *n*, %	2628.00	44.27	572.00	43.43	2056.00	44.51		1035.00	16.40	507.00	17.70	528.00	15.32	
Hodgkin’s disease, *n*, %	206.00	3.47	43.00	3.26	163.00	3.53		-	-	-	-	-	-	
Multiple myeloma, immune system malignant neoplasm, *n*, %	2989.00	50.35	675.00	51.25	2314.00	50.10		-	-	-	-	-	-	
Myelodysplastic syndrome, *n*, %	-	-	-	-	-	-		1036.00	16.41	486.00	16.96	550.00	15.96	
Myeloproliferative tumour, *n*, %	-	-	-	-	-	-		311.00	4.93	144.00	5.03	167.00	4.84	
Aplastic anaemia, *n*, %	-	-	-	-	-	-		133.00	2.11	53.00	1.85	80.00	2.32	
Others, *n*, %	2.00	0.03	0.00	0.00	2.00	0.04		138.00	2.19	60.00	2.09	78.00	2.26	
《Hospital factor》														
Academic hospital	2126.00	35.82	532.00	40.39	1594.00	34.51	<.01	3266.00	51.74	1338.00	46.70	1928.00	55.93	<.01
Attached dental clinic	4319.00	72.76	1025.00	77.83	3294.00	71.31	<.01	4865.00	77.08	2242.00	78.25	2623.00	76.10	.04
Cancer-based hospital	3662.00	61.69	800.00	60.74	2862.00	61.96	.08	4026.00	63.78	1752.00	61.15	2274.00	65.97	<.01
Case volume (number of hospital cases)	∼						<.01							<.01
∼Q2 (1-28 cases)	678.00	11.42	118.00	8.96	1219.00	26.39		271.00	4.29	58.00	2.02	213.00	6.18	
Q3 (29-67 cases)	662.00	11.15	311.00	23.61	1358.00	29.40		1483.00	23.49	575.00	20.07	908.00	26.34	
Q4 (68-277 cases)	4596.00	77.43	888.00	67.43	2042.00	44.21		4558.00	72.21	2232.00	77.91	2326.00	67.48	
Case volume (autologous/allogeneic)‡							<.01							<.01
∼Q2	2833.00	47.73	451.00	34.24	2382.00	51.57		1167.00	18.49	320.00	11.17	847.00	24.57	
Q3	2322.00	39.12	571.00	43.36	1751.00	37.91		2737.00	43.36	1316.00	45.93	1421.00	41.22	
Q4	781.00	13.16	295.00	22.40	486.00	10.52		2408.00	38.15	1229.00	42.90	1179.00	34.20	
Certification standard class							<.01							<.01
No certification	1792.00	30.19	175.00	13.29	1617.00	35.01		415.00	6.57	70.00	2.44	345.00	10.01	
Class 1	384.00	6.47	20.00	1.52	364.00	7.88		327.00	5.18	105.00	3.66	222.00	6.44	
Class 2	759.00	12.79	144.00	10.93	615.00	13.31		802.00	12.71	219.00	7.64	583.00	16.91	
Class 3	3001.00	50.56	978.00	74.26	2023.00	43.80		4768.00	75.54	2471.00	86.25	2297.00	66.64	
《Outcome》														
Length of stay, mean, SD	36.20	17.72	35.53	16.12	36.39	18.15	.69	89.23	40.44	86.35	37.96	91.63	42.25	<.01
In-hospital death	53.00	0.89	9.00	0.68	44.00	0.95	.66	966.00	15.30	411.00	14.35	555.00	16.10	.03
Antibiotic usage 30 d after transplantation, DDD	26.05	20.44	26.37	20.67	25.96	20.38	.64	72.42	42.39	69.13	40.81	75.16	43.47	<.01
Antibiotic usage 30 d after transplantation (broad), DDD	19.24	15.55	20.26	16.23	18.95	15.34	.02	46.93	30.11	45.70	29.63	47.95	30.48	.01
Antibiotic usage 14 d after transplantation, DDD	20.19	14.05	20.21	13.90	20.18	14.09	.96	35.34	20.92	34.10	20.35	36.37	21.33	<.01
Antibiotic usage 14 d after transplantation (broad), DDD	15.26	11.32	15.90	11.50	15.08	11.27	.02	24.19	15.79	23.87	15.82	24.45	15.76	.10
Narcotic usage 30 d after transplantation, DDD	0.26	1.10	0.28	1.18	0.25	1.07	.56	2.58	5.57	2.30	5.20	2.82	5.85	<.01
Narcotic usage 14 d after transplantation, DDD	0.23	0.99	0.26	1.07	0.22	0.97	.43	1.41	3.07	1.29	2.92	1.50	3.19	.01

⁎ Continuous variable: Mann–Whitney *U* test, discrete variable: *χ*^2^ test.

† CCI: QUAN score.

‡ Case volume: Autologous ∼Q2 (-15 cases), Q3 (16-27 cases), Q4 (28-41 cases) Allogeneic ∼Q2 (-7 cases), Q3 (8-33 cases), Q4 (34-57 cases).

As illustrated in Table 1, the oral and nonoral management groups for each of the autologous and allogeneic HSCT patients showed differences in the distributions of some variables when the influence of other variables was disregarded (ie, univariable analyses). Significant differences were observed in the numbers of broad-spectrum antibiotics used 14 and 30 days after transplantation in the autologous HSCT group. In the allogeneic HSCT group, significant differences were observed in the numbers of total antibiotics, broad-spectrum antibiotics, and narcotics used on days 14 and 30 post-transplantation. The nonoral management group tended to be utilizing more drugs (Table 1).

Multivariable analysis revealed that oral management had no significant effect on the total number of antibiotics used within 14 and 30 days after transplantation, the number of broad-spectrum antibiotics utilized, or any other outcome variable for patients undergoing autologous HSCT (Table 2).

**Table 2:** Multiple regression analysis of the impact of oral management on outcomes.

Outcome variable*	Partial regression coefficient			Standardized partial regression coefficient *β*	*P*
	B	95% CI (lower, upper)			
Autologous HSCT *N* = 5936					
Antibiotic usage					
Within 14 d after transplantation, DDD	–0.85	–1.73	0.03	–0.03	.06
Within 14 d after transplantation, DDD (broad)	0.47	–0.24	1.18	0.02	.20
Within 30 d after transplantation, DDD	–0.77	–2.05	0.52	–0.02	.24
Within 30 d after transplantation, DDD (broad)	0.78	–0.20	1.76	0.02	.12
Narcotic usage					
Within 14 d after transplantation, DDD	0.01	–0.10	0.10	0.00	.93
Within 30 d after transplantation, DDD	0.01	–0.06	0.08	0.00	.79
Length of stay (Log)	0.00	–0.01	0.00	–0.01	.97
Allogeneic HSCT *N* = 6312					
Antibiotic usage					
Within 14 d after transplantation, DDD	–2.66	–3.72	–1.60	–0.06	<.01
Within 14 d after transplantation, DDD (broad)	–0.78	–1.57	0.02	–0.03	.06
Within 30 d after transplantation, DDD	–6.74	–8.90	–4.58	–0.08	<.01
Within 30 d after transplantation, DDD (broad)	–2.79	–4.31	–1.27	–0.05	<.01
Narcotic usage					
Within 14 d after transplantation, DDD	–0.34	–0.49	–0.18	–0.05	<.01
Within 30 d after transplantation, DDD	–0.70	–0.99	–0.42	–0.06	<.01
Length of stay (Log)	0.00	–0.01	0.01	–0.01	.57

Independent variables: oral management, patient factors (adjusted for age, sex, CCI, BMI, ADL admission date, and TBI), and hospital factors (academic hospital, case volume, dental treatment available, and cancer-based hospital).

⁎ Outcome variable: antibiotic or narcotic use (DDD).

In patients undergoing allogeneic HSCT, when we adjusted for relevant patient factors and hospital factors (Table 2), the total number of antibiotics used within 14 days after transplantation was 2.66 DDD lower with oral management (*P* < .01), whereas that used within 30 days post-transplantation was 6.74 DDD lower (*P* < .01). Moreover, the quantity of broad-spectrum antibiotics administered within 30 days after transplantation was 2.79 DDD lower (*P* < .01). Additionally, the number of injectable narcotics used within 14 days after transplantation was 0.34 DDD lower (*P* < .01), whereas that used within 30 days post-transplantation was 0.70 DDD lower (*P* < .01). These findings suggest that implementing oral care resulted in less antibiotic and injectable drug use among allogeneic HSCT patients (Table 2). When generalized estimating equation analysis was conducted considering the influence of the hospital, results similar to those of the multiple regression analysis were obtained (Table S2). Logistic regression analysis was performed for in-hospital mortality. The analysis revealed that the implementation of oral management was associated with in-hospital mortality (odds ratio = 0.79, *P* < .01) (Table 3, Table S3).

**Table 3:** Logistic regression analysis of the impact of allogeneic HSCT on in-hospital death (*n* = 6312).

	Odds ratio	95% CI		*P*
		Lower	Upper	
Oral management	0.79	0.68	0.92	<.01
Male	0.72	0.62	0.83	<.01
Age	1.03	1.03	1.04	<.01
Transfer (admission date) Ref: Fully assisted				<.01
Partly assisted	1.35	0.45	4.06	.59
Without assistance	0.26	0.11	0.64	.00
Academic hospital	0.65	0.56	0.76	<.01
Attached dental clinic	1.16	0.97	1.38	.11
Cancer-based hospital	0.98	0.85	1.14	.80
Case volume (allogeneic) Ref: ∼Q2				.33
Q3	1.16	0.93	1.43	.19
Q4	1.19	0.94	1.50	.15
Certification standard class (Ref: No certification)				.26
Class 1	1.44	0.96	2.18	.08
Class 2	1.38	0.97	1.95	.07
Class 3	1.25	0.92	1.72	.16
CCI Score (include main disease) Ref: 0				
1	1.17	0.76	1.78	.48
2	1.16	1.00	1.35	.06
3	1.10	0.69	1.75	.70
4	1.28	0.80	2.03	.30
5	2.25	0.79	6.46	.13
6	2.25	0.84	5.98	.11
7	22.24	2.20	224.95	.01
≥8	1.13	0.46	2.77	.79
Total body irradiation	0.89	0.77	1.03	.11
(Constant)	0.13			<.01
Nagelkerke R2				.07
Hosmer and Lemeshow test				.20

Table 4 presents the implementation rates of oral management for patients undergoing HSCT categorized by the medical care delivery system. The certification standard for conducting unrelated HSCT has emerged as a key determinant influencing the implementation rate. Specifically, the implementation rate was observed to increase by 8.41% (*β* = 8.41, *P* < .01) for every class elevation (Table 5).

**Table 4:** Oral management implementation rate by hospital factor (*N* = 298).

	Hospital		Implementation rate (%)		*P*
	N	%	Mean	SD	
Case volume					<.01
∼Q2 (1-28 cases)	149	50.0	9.29	22.49	
Q3 (29-67 cases)	76	25.5	25.20	30.65	
Q4 (68-277 cases)	73	24.5	39.74	32.08	
Cancer-based hospital					<.01
Applicable	81	27.2	13.19	26.12	
Not applicable	217	72.8	23.65	30.87	
Attached dental clinic					<.01
No	82	27.5	13.69	27.06	
Yes	216	72.5	23.51	30.64	
Bed size					<.01
<200	7	2.3	0.00	0.00	
200-399	40	13.4	14.58	28.25	
400-599	119	39.9	16.24	27.20	
600-799	75	25.2	28.60	31.06	
≥800	52	17.4	29.62	34.07	
Unknown	5	1.7	0.00	0.00	
Certification standard class					<.01
No certification	144	48.3	7.53	18.45	
Class 1	101	33.9	40.88	33.51	
Class 2	29	9.7	23.29	31.14	
Class 3	24	8.1	12.96	21.83	
Academic hospital					<.01
No	223	74.8	18.18	29.11	
Yes	75	25.2	28.62	31.33

**Table 5:** Multiple regression analysis of hospital factors influencing oral care implementation rates (forced entry) (*N* = 298).

	Partial regression coefficient			Standardized partial regression coefficient *β*	*P*	VIF
	*Β*	95% CI (lower, upper)				
Certification standard class*	8.41	5.39	11.44	0.38	<.01	1.90
Bed size	–0.01	–0.02	0.01	–0.06	.34	1.50
Cancer-based hospital	1.76	–5.88	9.40	0.03	.65	1.26
Attached dental clinic	6.08	–0.76	12.92	0.09	.08	1.04
Number of allogeneic HSCT patients	0.18	0.04	0.32	0.18	.01	1.92
(Constant)	5.45	–4.13	15.02		.26	

⁎ 0 = No certification, 1 = Class 1, 2 = Class 2, 3 = Class 3.

## Discussion

Patients undergoing HSCT are particularly susceptible to fatal infections, primarily due to leukopenia resulting from bone marrow suppression induced by whole-body radiotherapy and the administration of high-dose chemotherapy. The occurrence of graft-versus-host disease post-transplantation requires immunosuppressive therapy, which also affects the course of infectious diseases. Given the importance of infection control, our study demonstrates the importance of oral management as a preventive measure against infection. We conducted a comparative analysis of antibiotic use and in-hospital mortality rates based on the implementation of oral management before and after transplantation. The findings demonstrated that oral care pre- and post-transplantation plays a crucial role in reducing the number of antibiotics administered within 14 and 30 days post-transplantation and mitigating in-hospital mortality rates. BSI often originates from oral bacterial cultures.^27^ Our findings indicated that oral management before and after transplantation effectively mitigates the occurrence and severity of life-threatening bacterial infections during the leukopenic period after HSCT. Concerning in-hospital mortality among patients undergoing allogeneic HSCT, an odds ratio of 0.79 associated with the implementation of oral management is a noteworthy result.

The incidence of OM in autologous or allogeneic HSCT is high, at 70% to 99%.^9^ OM poses significant complications for patients undergoing HSCT, encompassing issues such as infection susceptibility, severe pain, and disruptions in eating patterns.^13^^,^^28^^,^^29^ Additionally, increased severity of OM correlates with a prolonged length of hospital stays.^30^ Therefore, oral management cantered on dentists and dental hygienists is recommended, including dental treatment before and after transplantation.^31^, ^32^, ^33^, ^34^, ^35^, ^36^ In Japan, the ‘Severe Side Effect Disease-Specific Response Manual: Stomatitis Due to Anticancer Drugs’ does not describe oral mucosal disorders related to HSCT.^32^ European and American research entities have highlighted that one obstacle in formulating an effective oral care regimen to mitigate oral complications in patients undergoing HSCT stems from inadequate evidence to assess such complications, owing to variances in conditioning regimens. Consequently, an international multicentre comparative study was conducted to address this gap.^19^

Furthermore, this study revealed that oral management before and after transplantation contributed to a reduction in narcotic use within 14 and 30 days post-transplantation. These findings are consistent with those of previous studies.^12^, ^13^, ^14^^,^^32^ Vagliano et al^37^ reported that 71.4% of patients undergoing HSCT developed OM, with 21.6% experiencing grade 3 or higher OM. Grade 3 OM requires the consumption of only liquid food, whereas grade 4 OM renders eating impossible. Narcotic analgesics may be used for pain management in patients with grade 3 or higher OM.^38^ In our study, we observed that the narcotic dosage was reduced in cases where pre- and post-transplant oral management was conducted. These findings imply a correlation with avoidance of severe OM.^16^

Thus, maintaining optimal oral health before and after transplantation through interdisciplinary collaboration among experts for patients undergoing HSCT mitigates OM and infections and enhances patients’ quality of life. However, upon closer examination at the facility level, the implementation of oral care before and after transplantation was suboptimal, with variations based on the function of the hospital (medical care delivery system). The accreditation standards established by the Japanese Society for Transplantation and Cellular Therapy play a pivotal role in the implementation of oral care practices. When analysing facilities that did not meet the standards vs those that met standards 1 to 3, an approximately 8% increase was observed in the oral care implementation rate for each higher standard. The highest facility standard mandates the provision of medical care for transplants and the performance of six new allogeneic HSCTs within the past 12 months. Additionally, specifications regarding the number of cases and the composition of the transplantation team, including physicians, nurses, and HCTC personnel, exist.^39^ High-volume hospitals may accept critically ill patients.^40^ Furthermore, the high implementation rate observed in high-volume hospitals is hypothesized to result from the tailored provision of oral management before and after transplantation based on individual patient needs. Concerning the implementation rate, further investigation is warranted to determine whether enhancements are possible at each hospital or if the outcomes reflect appropriate implementation for patients requiring such care. In Japan, the establishment of regional base hospitals to facilitate HSCT has been underway since 2019 as part of a national policy to ensure accessibility to HSCT irrespective of patients’ geographic locations. However, as patients in need of HSCT are distributed across the country, disseminating expertise from core hospitals to smaller-scale facilities is imperative.^41^

Through collaborative efforts with a multidisciplinary team comprising dentists and dental hygienists, a conducive oral environment for patients undergoing HSCT before and after transplantation must be promoted. These practices culminate in an enhanced quality of life for transplant recipients, reflected in reduced infection rates, lower incidences of sepsis and mortality, along with effective pain management. Moreover, such initiatives can yield favourable economic outcomes.

In Japan, reduction in OM through systematic oral care interventions for patients with leukaemia and HSCT has been documented. However, these studies were conducted on a small scale.^15^, ^16^, ^17^^,^^42^ Since 2016, the evaluation of oral management before and after transplantation has been facilitated by assessing medical fees, enabling large-scale database research that utilizes medical performance data. We consider it significant that we were able to elucidate the effects of implementing oral management using this approach.

Owing to the utilization of clinical practice data, this study had four primary limitations:

1. Oral conditions before transplantation or the actual dental interventions performed were not considered. The DPC data do not include data on oral conditions; therefore, adjusting for oral conditions that may affect mucosal damage and infection was impossible. Additionally, the timing of the initiation of dental interventions was not considered. In the future, by combining the DPC data used in this study with dental clinical performance data, it may be possible to conduct analyses that also consider this factor.
2. Differences in conditioning regimens were not considered. The state of immunosuppression and mucosal damage varies greatly depending on the conditioning treatment before transplantation (RIC, MAC). In this study, we were unable to risk-adjust for these differences.
3. Adjustment for disease and patient conditions was insufficient. The risk of HSCT treatment-related mortality needs to be assessed to evaluate in-hospital mortality. A representative score is the Hematopoietic Cell Transplantation-specific Comorbidity Index.^43^ Although patients with a high Hematopoietic Cell Transplantation-specific Comorbidity Index score were not enrolled, we did not have the necessary information to measure this score.
4. Our study did not account for possible selection bias of patients who received oral management. However, as noted in previous studies, physicians are not oral health specialists and therefore do not evaluate the oral condition to order dental interventions.^44^ Consequently, we believe that the requests for oral management by physicians for HSCT patients are likely based on their understanding of the usefulness of oral management and the hospital’s policies, rather than the actual oral condition of the patients.

However, this study does have a certain level of significance. Given the difficulty in establishing a database that links dental and medical treatment data and conducting prospective studies, the results of a study with a large sample size using DPC data would be extremely significant. Additionally, adjusting for patient background factors (disease factors), which are crucial for evaluating patient outcomes, is extremely challenging.^45^ Our study’s strength lies in considering this difficulty and evaluating oral management while adjusting for patient background factors to the extent possible.

Despite the aforementioned limitations, the involvement of specialists, particularly dentists and dental hygienists, in assessing the condition of the oral cavity before and after transplantation and in administering tailored care accordingly holds significant value. This approach has contributed to reducing infections, alleviating pain, and mitigating eating disorders in patients while suppressing the severity of these complications. Validating these effects through a prospective study that comprehensively incorporates these adjustments is warranted.

Ensuring an appropriate oral environment for patients undergoing allogeneic HSCT before and after transplantation can play a pivotal role in preventing infection, extending life expectancy, and alleviating pain. Establishing a medical care delivery system that fosters collaboration between dental and medical teams is essential for providing patients undergoing HSCT with safe, suitable, and high-quality medical care.

## Author contributions

Mutsuko Moriwaki: Writing – original draft, conceptualization, data curation, formal analysis, investigation, methodology, project administration, visualization. Mikayo Toba: Writing – original draft, conceptualization, data curation, investigation, methodology, project administration. Makiko Takizawa, Hiroaki Shimizu, and Shinobu Imai: Writing – review and editing, supervision. Haruna Tanaka: Data curation, formal analysis. Chihiro Takahashi: Data curation. Masayuki Kakehashi: Writing – review and editing, formal analysis, supervision. Kiyohide Fushimi: Writing – review and editing, funding acquisition, supervision.

## Funding

This work was supported by a Grant-in-Aid for Scientific Research from the Ministry of Education, Culture, Sports, Science and Technology of Japan (Grant No. 18K09996, 23K27871). The sponsor was not involved in study design; in the collection, analysis, and interpretation of data; in the writing of the report; or in the decision to submit the article for publication.

## Data availability

The datasets generated and/or analysed during the current study are not publicly available because of contracts with hospitals providing data to the database but are available from the corresponding author (K.F.) on reasonable request.

## Conflict of interest

The authors declare that they have no known competing financial interests or personal relationships that could have appeared to influence the work reported in this article.

## Appendix Supplementary materials

Image, application 1

## Footnotes

Supplementary material associated with this article can be found in the online version at doi:10.1016/j.identj.2025.04.003.
