# Supplementary material for: Oral Management Improves Patient Outcomes in Hematopoietic Stem Cell Transplantation
Source: Int Dent J. 2025 May 9;75(4):100822. doi: 10.1016/j.identj.2025.04.003 (PMC12139409; doi:10.1016/j.identj.2025.04.003)
Supplement: Supplementary file 1 [file mmc1.docx]

**Supplementary Material**

**Supplementary Table S1** Drug (ACTcode) list

| Drug classification | ATC code name | Abbreviations | ACTCODE |
| --- | --- | --- | --- |
| Narcotic (injection) | Fentanyl | - | N01AH01 |
|  | Hydromorphone | - | N02AA03 |
|  | Morphine | - | N02AA01 |
|  | Oxycodone | - | N02AA05 |
|  | Pethidine | - | N02AB02 |
|  | Pethidine, combinations excl. psycholeptics | - | N02AB52 |
|  | Remifentanil | - | N01AH06 |
| Antibacterial Agents (broad spectrum) (injection) | Faropenem | FRPM | J01DI03 |
|  | Ciprofloxacin | CPFX | J01MA02 |
|  | Imipenem/Cilastatin | IPM/CS | J01DH51 |
|  | Cefpirome | CPR | J01DE02 |
|  | Doripenem | DRPM | J01DH04 |
|  | Meropenem | MEPM | J01DH02 |
|  | Cefepime | CFPM | J01DE01 |
|  | Levofloxacin | LVFX | J01MA12 |
|  | Tazobactam/Piperacillin | TAZ/PIPC | J01CR05 |
|  | Relebactam/Imipenem/Cilastatin | REL/IPM/CS | J01DH56 |
|  | Cefozopran | CZOP | J01DE03 |
|  | Panipenem/Betamipron | PAPM/BP | J01DH55 |
|  | Pazufloxacin | PZFX | J01MA18 |
|  | Tebipenem Pivoxil | TBPM-PI | J01DH06 |
|  | Biapenem | BIPM | J01DH05 |
|  | Lascufloxacin | LSFX | J01MA25 |

**Supplementary Table S2** Results of regression analyses for the impact of oral management on the outcomes for patients undergoing allogeneic HSCT (Generalized estimating equation; GEE)

N＝6,132

| Outcome Variable | | B | 95%CI | | P value |
| --- | --- | --- | --- | --- | --- |
|  |  |  | Lower | Upper |  |
| Antibiotic usage | |  |  |  |  |
|  | within 14 days after transplantation, DDD | -1.66 | -3.20 | -0.12 | 0.03 |
|  | within 14 days after transplantation, DDD (broad) | -0.26 | -1.31 | 0.80 | 0.63 |
|  | within 30 days after transplantation, DDD | -4.49 | -7.46 | -1.52 | 0.00 |
|  | within 30 days after transplantation, DDD (broad) | -1.66 | -3.20 | -0.12 | 0.03 |
| Narcotic usage | |  |  |  |  |
|  | within 14 days after transplantation, DDD | -0.25 | -0.45 | -0.05 | 0.01 |
|  | within 30 days after transplantation, DDD | -0.54 | -0.92 | -0.15 | 0.01 |

1) Nested within hospitals

2) Independent variable: Oral management, patient factor (Adjusted for age, sex, CCI, BMI, length of stay, ADL admission date, and TBI), and hospital factor (Academic Hospital, case volume, dental treatment available, and cancer base hospital)

**Supplementary Table S3** Results of regression analyses for the impact of oral management on in-hospital death of patients undergoing allogeneic HSCT (Generalized estimating equation; GEE)

N＝6,132

| Outcome Variable | Odds | B | 95%CI | | P value |
| --- | --- | --- | --- | --- | --- |
|  |  |  | Lower | Upper |  |
| Hospital in death | 0.81 | -0.21 | -0.39 | -0.03 | 0.02 |

1) Nested within hospitals

2) Independent variable: Oral management, Patient’s factor (Adjusted for Age, sex, CCI, BMI, length of stay, ADL admission date, and TBI), and Hospital factor (Academic Hospital, Case Volume, Dental treatment available, and Cancer base hospital)
